# In assisted reproduction by IVF or ICSI, the rate at which embryos develop to the blastocyst stage is influenced by the fertilization method used: a split IVF/ICSI study

**DOI:** 10.1007/s10815-018-1358-3

**Published:** 2019-01-09

**Authors:** Barbara Speyer, Helen O’Neill, Wael Saab, Srividya Seshadri, Suzanne Cawood, Carleen Heath, Matthew Gaunt, Paul Serhal

**Affiliations:** 1 Institute for Women’s Health, University College London, London, UK; 2 Centre for Reproductive and Genetic Health, London, UK

**Keywords:** IVF, ICSI, Embryo development

## Abstract

**Purpose:**

To compare in vitro fertilization (IVF) with intracytoplasmic sperm injection (ICSI) in regard to post-fertilization development and outcome with the purpose of ascertaining the most effective fertilization method for assisted reproduction.

**Methods:**

A retrospective cohort study of 136 split IVF/ICSI cycles (where sibling oocytes are fertilized by two different methods using the same sperm sample).

**Results:**

IVF-derived embryos developed to the blastocyst stage at a significantly faster rate than ICSI-derived embryos. There was no significant difference in fertilization or livebirth rates between the two fertilization methods.

**Conclusions:**

For patients with sperm progressive motility ≥ 1.0 × 10^6^/ml (who usually constitute the majority of patients), no significant difference between the two fertilization methods was found in regard to fertilization rate or livebirth rate. Remaining factors influencing choice between the two methods appear to be restricted to convenience, financial considerations and concern with regard to possible perpetuation of genetically linked infertility to future generations.

## Introduction

In the field of assisted reproduction, the introduction of intracytoplasmic sperm injection (ICSI) [16] has resulted in a choice of fertilization method between conventional in vitro fertilization by insemination (IVF) and fertilization by ICSI. Fertilization by insemination calls upon functions of the sperm that are bypassed by injection directly into the oocyte. Therefore, the choice of the fertilization method for an individual couple should largely depend upon the results of the sperm analysis. Severe oligospermia (low sperm concentration), asthenozoospermia (low motility) or teratozoospermia (abnormal morphology) indicate the use of ICSI. Another factor to be considered is the reproductive history of the couple. Previous successes or failures using either IVF or ICSI are obviously relevant to future treatment. In the case of IVF, unexpected complete fertilization failure (CFF) in an individual cycle is a well-known phenomenon and is a risk to the success of IVF cycles. Opinion is divided as to whether these isolated occurrences necessitate the universal use of ICSI in assisted reproduction. There remains much support for the view that IVF has a role when the sperm analysis shows no severe deficiencies and where there is no history of previous CFF [2, 6, 9].

When there is uncertainty as to which fertilization method to use, the option of split IVF/ICSI can be adopted. In this method, approximately half of the sibling oocytes are randomly chosen to be fertilized by IVF and the remainder are fertilized by ICSI. Comparing the fate of multiple oocytes from the same cycle using the same sperm sample gives a valid comparison between fertilization methods, helping to plan the future treatment of the couple.

Some relatively early studies [1, 11] used split cycles mainly for the purpose of comparing IVF and ICSI fertilization rates, giving much attention to their use as a safeguard against CFF. In many cases, it may have been assumed that once the oocyte was fertilized, the method of fertilization ceased to be relevant. However, the two fertilization methods are so different [12] that the possibility of differences in outcome cannot be ignored. Vitek et al. [22] compared the three different fertilization procedures: all-IVF, all-ICSI and split IVF/ICSI and, in support of Bhattacharya et al. [2], found no significant difference in fertilization rate between IVF and ICSI. For a single cycle, split IVF/ICSI and all-ICSI were each associated with a 3.0 percentage higher livebirth rate when compared with all-IVF. The authors reached the conclusion that, for a single cycle, all-ICSI was not justified due to the higher cost per livebirth.

The present study aims, through the use of split IVF/ICSI cycles, to explore the choice between the two main fertilization methods with a view to finding whether or in what way fertilization method affects post-fertilization embryo development. Particular attention was given to rates of post-fertilization development and livebirth outcome. A record was kept of the history of each embryo, enabling comparison of the state and outcome of embryos both before and after transfer to the uterus.

## Methods

### Patients

This is a retrospective cohort study of the results of 136 split IVF/ICSI cycles performed at the Centre for Reproduction and Genetic Health (CRGH), London W1W 5QS between 2004 and 2018. The study was approved by the University College London Ethics Committee, ethical approval number 05/Q0502/143. All 136 patients in the study gave written consent and were attending the CRGH with problems of infertility. Each cycle included the transfer of either one or two fresh embryos into the female patient’s uterus. Female patients were < 43 years of age. All male patients had percentage motility ≥ 27 and a sperm progressive motility concentration of ≥ 1.0×10 ^6^ /ml.

Couples who required gamete donation or cases where either the male or female was found to have an abnormal blood karyotype were excluded from the study.

### Sperm preparation

After liquefaction and semen analysis, the semen sample was centrifuged at 300*g* for 20 min in a 45% and 90% PureSperm density gradient (Nidacon International, Gothenburg, Sweden). Dilutions were made with fertilization medium (Quinn’s Advantage Fertilization (HTF) Medium, CooperSurgical, Trumbull, CT, USA). The pellet was washed twice with 10-min centrifugation each time and suspended in fertilization medium. The sperm concentration was determined, and the suspension was kept in an incubator at 37 °C with a 6% carbon dioxide atmosphere in preparation for IVF and ICSI.

### IVF and ICSI procedures

The stimulation protocol has been described previously (Speyer et al. 2010) [19]. When at least two leading follicles reached the diameter of 18 mm, hCG was administered by injection. Dishes for oocyte culture were prepared on the day of hCG injection by placing 0.5 ml of fertilization medium into each well of 5-well Nunc dishes (Fisher Scientific UK Ltd., Loughborough, UK) overlaid with 0.5 ml Ovoil (Vitrolife, Gothenburg, Sweden). The dishes were kept at 37 °C with 6% CO_2_ to be ready for oocyte retrieval, which was done 37 h after hCG. Each retrieved cumulus-oocyte complex was placed in the medium in a well. The resulting wells were in two groups destined for IVF and ICSI respectively. The numbers in the two groups were made as equal as possible without discarding oocytes. In 82% of cycles, the two numbers differed by only one oocyte.

### IVF insemination

Incubation of the oocytes destined for IVF continued at 37 °C and 6% CO_2_ before and after insemination, which was performed 40 h after hCG. A volume of prepared sperm suspension sufficient to give a final concentration of 200,000 motile sperm/ml was added to each well containing a cumulus-oocyte complex. Incubation under the same conditions continued until 18–20 h after insemination, when fertilization checks were done. Fertilized embryos were transferred to SAGE 1-step medium with HSA and phenol red (CooperSurgical) and incubated at 37 °C with 6% CO_2_.

### ICSI insemination

Incubation of the cumulus-oocyte complexes in the wells of the Nunc dishes was continued in fertilization medium until denudation, which was done 41 h after hCG. Denudation was achieved through the use of cumulase (CooperSurgical, USA). ICSI was then done by standard procedures [5, 16]. The prolonged incubation of the cumulus-oocyte complexes before denudation gave the oocytes time to mature [23]. Even so, it was found after denudation that a small percentage of oocytes (34 oocytes in total from 136 cycles) were still immature, and these were termed “poorly maturing oocytes”. Since these oocytes could not be fertilized they had to be discarded. A similar percentage of such oocytes was observed at the fertilization check of the oocytes that had been subjected to the IVF insemination process. Sperm-injected oocytes were placed into SAGE 1-step medium with HSA and phenol red and incubated at 37 °C with 6% CO_2_. Fertilization checks were performed 15.5–17.5 h after sperm injection. Incubation of fertilized embryos continued in the SAGE medium.

### Comparison of the incubation stages of IVF and ICSI

Both methods started with retrieved oocyte-cumulus complexes incubated in fertilization medium. In the case of IVF, fertilization occurred in the same medium (beginning 40 h after hCG) and ending at fertilization check (58–60 h after hCG). Transfer of zygotes from fertilization medium to Sage medium occurred at fertilization check. In the case of ICSI, fertilization and transfer to SAGE medium both occurred 41 h after hCG. With both methods, culture in SAGE medium continued until optimum embryo development was reached around days 3–7 after fertilization.

### Embryo transfer

Either one or two embryos were transferred, depending upon the fertility and informed wishes of the patient. Fertilized embryos were carefully inspected at the time of optimum development. The embryos for transfer were chosen according to their grading quality and state. Blastocysts, where present, were chosen for transfer rather than earlier stages of development. Blastocysts were graded using the Cornell grading system [14] and the best-quality ones chosen for transfer, regardless of whether they were originally from IVF or ICSI. This meant that in some cases (mixed transfers), one IVF and one ICSI embryo were transferred together. Any good-quality embryos not required were cryopreserved for possible use in subsequent freeze/thawed embryo transfers.

### Statistics

The SPSS software version 24 was used for comparison of means by independent samples *t* test. For non-normal data (Tables 2 and 3) the Mann-Whitney test was used.

## Results

Table 1 gives information on the 136 couples in the study, the reasons for their infertility and the main features of their sperm analyses. The diagnosis of male factor infertility was given if the sperm analysis showed a sperm concentration of < 18 million/ml or a progressive motility of < 40%. Although it is more usual to use only the ICSI method on male factor patients, use of a split cycle gives the chance of success to both fertilization methods.

**Table 1:** Clinical data on patients

Data on patients	
No. of couples	136
No. of cycles	136
Average female age (± SD*)*	35.0 (± 4.0)
Average male age (± SD*)*	39.2 (± 6.2)
Diagnosis	
Male factor	27.9
Tubal disease	10.3
Ovulatory disorder	5.1
Endometriosis	5.1
Other uterine disorders	5.9
Polycystic ovaries	8.1
Unexplained	37.5
Data on sperm	
Mean sperm conc. × 10^6^/ml*.* (± SD*)*	31.4 (± 22.0)
Mean percentage motility (± SD*)*	55.3 (± 13.1)
Mean normal morphology	16.7 (± 12.4)

Table 2 shows mean fertilization rates for the 136 split cycles. There was no significant difference between IVF and ICSI in fertilization rate whether or not the cycles with complete IVF fertilization failure (CFF) were omitted from the calculation. The omission of these 4 cycles was found to slightly increase the mean fertilization rate for ICSI as well as increasing that for IVF. This is explained by the fact that two of the 4 CFF cycles had a low (25% and 33%) fertilization rate using ICSI. It was concluded that the sperm defect in these two patients might be more extensive than in the other two patients with CFF and affect fertilization by ICSI as well as by IVF, and in fact, these two patients did not achieve a livebirth. The other two CFF patients, with ICSI fertilization rates 66.7% and 85.7%, achieved respectively a singleton and a twin livebirth. The 4 CFF cycles were included in the main study except where stated otherwise.

**Table 2:** Fertilization rates

Fertilization method	Mean fertilization rate (%)
IVF	66.5 (± 25.8)
ICSI	64.1 (± 22.2)
IVF excluding CFF cycles	68.5 (± 23.4)
ICSI excluding CFF cycles	64.4 (± 22.0)

Mean fertilization rates. The total number of split IVF/ICSI cycles is 136 and the number of cycles with complete IVF fertilization failure (CFF) is 4. The difference *P* between IVF and ICSI fertilization rates was non-significant regardless of whether or not the CFF cycles were included. The Mann-Whitney test confirmed non-significance between fertilization rates (*P* = 0.182)

It was of interest to investigate the fate of the fertilized embryos in order to detect any effect of the mode of fertilization upon subsequent outcomes such as suitability for embryo transfer to the female patient. In each of the 136 cycles, the best quality one or two embryos regardless of fertilization method were transferred. For each cycle, the fertilization method (or methods in the case of mixed transfers) of the transferred embryos was recorded. It was found that in 61(44.9%) of the 136 cycles, the transferred embryos were IVF-fertilized, in 41 cycles (30.1%) they were ICSI-fertilized and in the remaining 34 cycles (25.0%) (mixed transfers) one IVF-fertilized embryo and one ICSI-fertilized embryo were transferred together. The difference between the numbers of IVF-fertilized (61) and ICSI-fertilized (41), both out of 136 cycles, was significant by *t* test (*P* = 0.012).

In choosing embryos for transfer, it is the clinic’s policy to transfer blastocysts in preference to morula- or cleavage-stage embryos since they have been reported to yield higher pregnancy rates [3, 10]. Therefore, the next step was to compare the total numbers of IVF-derived and ICSI-derived blastocysts formed during the period of blastocyst formation, which ended around day 7 post-fertilization. The results are shown in Table 3. The total numbers of blastocysts formed were non-significantly higher for IVF fertilization (332) than for ICSI fertilization (287). This lack of significance between the total numbers of IVF-derived and ICSI-derived blastocysts formed raises the question as to why was the number of cycles in which IVF-fertilized embryos were transferred significantly higher than the number of cycles in which ICSI-fertilized embryos were transferred? In this respect, the total numbers of blastocysts from all 136 cycles shown in Table 3 does not give a reliable indication of the relative numbers of IVF-fertilized and ICSI-fertilized embryos transferred. The decisions as to which embryos to transfer were dealt with one cycle at a time. In some cycles, a large number of blastocysts were produced, and since only one or two embryos were transferred, many blastocysts were cryopreserved and lost to the calculation of total blastocyst number. Other cycles had no blastocysts, and the decision was based on the quality of less-developed embryos. Data on the embryos actually transferred gives a more direct picture of the embryos chosen for best developmental stage and quality, and this information is shown in Table 4. The total number of embryos transferred was 228. Of these, the number selected to be transferred as IVF-fertilized only or as ICSI-fertilized only was 94 and 66 respectively. The difference in numbers is significant (*P* = 0.006). If, instead of the number of embryos, we consider the number of blastocysts, we see that Table 4 includes 80 IVF-derived blastocysts, 51 ICSI-derived blastocysts, and 39 mixed blastocysts. Of the mixed transferred blastocysts, 20 were IVF-derived and 19 ICSI-derived. Including these blastocysts, the total number of IVF-derived and ICSI-derived transferred blastocysts respectively was 80 + 20 and 51 + 19, i.e. 100 and 70. The difference between 100 and 70, both out of 228 is significant (*P* = 0.004). In mixed transfers, the number of IVF-derived and ICSI-derived embryos for each stage of development was equal or close to equal. Total embryo count was 34 for each fertilization method in the mixed column. The number of morulas was 2 for IVF-derived and 3 for ICSI-derived. Of the 20 mixed cleavage-stage embryos, 10 were derived from each fertilization method. The high percentage of cleavage stage embryos in the mixed column (Table 4) was associated with a relatively low level of livebirth events for this column (Table 5).

**Table 3:** Blastocyst formation in 136 split ivf/icsi cycles

	IVF	ICSI	Significance *P*
Total number of embryos	665	611	NS
Total number of blastocysts	332 (49.9%)	287 (47.0%)	NS
Average number of blastocysts per cycle	2.44	2.11	NS

The total number of blastocysts formed after each fertilization method up to and including the seventh day after fertilization. The Mann-Whitney test confirmed non-significance between the number of blastocysts from the two different fertilization rates (*P* = 0.202)

**Table 4:** Embryos transferred In 136 SPLIT IVF/ICSI cycles

Developmental stage	IVF	ICSI	Mixed
Blastocysts	80 (85.1)	51 (77.3)	39 (57.4)
Morula	5 (5.3)	3 (4.5)	5 (7.4)
Pre-morula	0 (0)	2 (3.0)	4 (5.9)
Cleavage	9 (9.6)	10 (15.2)	20 (29.4)
Total	94	66	68

The cycles in the table are the 136 split IVF/ICSI cycles listed in Table 1. Figures in brackets are percentages of the totals. All 228 embryos in the table were transferred. Either one or two embryos were transferred per cycle, depending upon the fertility and the informed wishes of the couple. The best embryos available in each cycle with regard to developmental stage and quality were chosen for transfer. The options for transfer were (1) IVF-derived embryos only, (2) ICSI-derived embryos only or (3) (mixed transfer) one IVF-derived embryo and one ICSI-derived embryo transferred together

In calculating the significant difference in the number of IVF-derived and ICSI-derived embryos transferred one can either omit or include the embryos of the mixed transfers. Of these 68 mixed embryos, 34 were IVF-derived and 34 ICSI-derived. Of the 39 mixed blastocysts, 20 were IVF-derived and 19 ICSI-derived
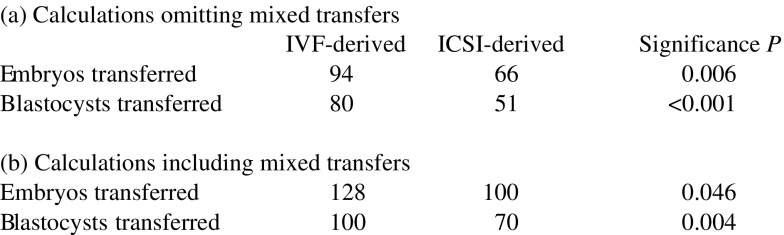

**Table 5:** Outcomes from 136 split IVF/ICSI cycles

Origin of transferred embryos	IVF-derived	ICSI-derived	Mixed
No. of cycles	61	41	34
No. of singleton live births	26 (42.6)	16 (39.0)	10 (29.4)
No. of twin live births	10 (16.4)*	10 (24.4)*	3 (8.8)
No. of biochemical pregnancies	4 (6.6)	2 (4.9)	4 (11.8)
No. of fetal heartbeat miscarriages	4 (6.6)	4 (9.8) ^◊^	2 (5.9)
No. of negative pregnancy cycles	17 (27.9)	9 (22.0)	15 (44.1)
Total live birth events	36 (59.0)	26 (63.4)	13 (38.2)

Embryo transfers in the table were from the 136 split IVF/ICSI cycles listed in Table 1. The transferred embryos originated from one of 3 options: IVF-derived only, ICSI-derived only, or the third option (mixed transfer) from one IVF-derived embryo together with one ICSI-derived embryo. The choice of embryos to transfer was based on developmental stage and quality. Livebirth events are the sum of singleton and twin livebirths. Figures in brackets are percentages of the totals. The differences between outcomes are as follows:
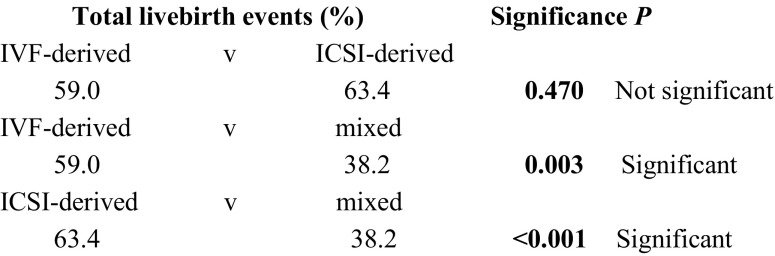

*One of the IVF-derived twin livebirths originated in one sac and resulted from the transfer of one blastocyst only. Two of the ICSI-derived twin livebirths and one of the IVF-derived twin livebirths each resulted from the transfer of two cleavage stage embryos

^◊^One of these was an ectopic pregnancy

The evidence from Table 4 indicates that the embryologists, in choosing one or two embryos for transfer to the female patient’s uterus, chose IVF-derived blastocysts significantly more often than they chose ICSI-derived blastocysts. An experienced embryologist judged the best time to carry out embryo transfer, which was when further incubation was unlikely to result in quality improvement. The preferred choice of IVF-derived blastocysts could be explained if IVF-derived embryos developed faster to the blastocyst stage than ICSI-derived embryos, yielding a better choice of blastocyst at the time of embryo transfer. To test this assumption, we counted the number of IVF-derived and ICSI-derived blastocysts recorded on the day of first blastocyst appearance. The 22 cycles in which neither fertilization method produced blastocysts and also the 27 cycles in which an equal number of IVF-derived and ICSI-derived blastocysts were recorded on the day of first blastocyst appearance were excluded from the calculation of significance. The calculation was performed firstly including the 4 CFF cycles and secondly excluding the 4 CFF cycles (see Table 6). Recorded details of the 136 cycles were examined and the origin of each blastocyst present on the day when the first blastocyst(s) appeared was noted. Lists were made of cycles in which IVF-derived blastocysts outnumbered ICSI-derived blastocysts and of cycles in which ICSI-derived blastocysts outnumbered IVF-derived blastocysts. Table 6 shows that when the 4 CFF cycles were excluded from the calculation, there was a significant difference (*P* = 0.012) in favor of IVF-derived blastocysts (on the day in which blastocysts were first observed) between the number of cycles in which IVF-derived blastocysts predominated and the number of cycles in which ICSI-derived blastocysts predominated. This indicates that under the conditions of the study, there was a significant difference in favor of IVF-derived embryos between IVF-derived and ICSI-derived embryos in the rate of development to the blastocyst stage. This observation explains the significantly greater number of IVF-derived embryos transferred in the 136 IVF/ICSI cycles compared to the number of ICSI-derived embryos. On the chosen day of transfer, IVF-derived blastocysts may have been the only blastocysts present or may have outnumbered ICSI-derived blastocysts, making it likely that they were chosen for transfer.

**Table 6:** Effect of including CFF cycles

	Total number of cycles	Number of cycles in which IVF-derived blastocysts predominated	Number of cycles in which ICSI-derived blastocysts predominated	Significance of difference *P*
CFF cycles included	136	51	36	0.051
CFF cycles excluded	132	51	32	0.012

The table shows the effect on the significance of fertilization method of adding 4 complete IVF fertilization failure cycles (CFF cycles) into a group of 132 non-CFF split IVF/ICSI cycles. For each cycle, the recorded number of IVF-derived and ICSI-derived blastocysts present on the day of first blastocyst appearance was made. The table was constructed from two lists of cycles, in the first of which the number of IVF-derived blastocysts exceeded that of ICSI-derived blastocysts and in the second of which the opposite occurred

The outcomes of the cycles that received IVF-derived embryos, ICSI-derived embryos and embryos from both methods of fertilization (mixed transfers) are shown in Table 5. The choice of whether to transfer one or two embryos to the patient largely depended upon the fertility of the patient, the less fertile patients being more likely to receive two embryos. For this reason, the outcomes in Table 5 pay no regard to whether one or two embryos were transferred. Statistical analysis showed no significant difference (*P* = 0.47) between total live birth events (59% and 63.4%) derived from IVF and ICSI fertilization respectively, although both were significantly higher than mixed transfers (*P* = 0.003 and < 0.001 respectively). Thus, there is no significant evidence from the data in Table 5 that either IVF or ICSI fertilization is superior to the other in terms of producing a livebirth.

The question arises as to how to choose between IVF and ICSI in situations where it may be inconvenient to carry out a split cycle. Often, a value is set for concentration or motility below which ICSI is always chosen as the fertilization method, but this method is not ideal because cut-off points for IVF fertilization need to be decided. The results from the present study show that 7 IVF-derived livebirths resulted from patients whose sperm concentration was 14–19 million per ml, and a frequent cut-off point is 20 million per ml, which would have excluded these IVF-derived livebirths, although they might have occurred if ICSI-derived embryos had been chosen for transfer.

## Discussion

In each cycle, the decision had to be made as to which embryos should be transferred to the female patient’s uterus. This was decided on the basis of the degree of development and quality of each embryo in the cycle. The number of cycles in which IVF-fertilized embryos were transferred was significantly higher than the number of cycles in which ICSI-fertilized embryos were transferred. This was especially noticeable in the significantly higher number of IVF-fertilized blastocysts transferred compared to the number of ICSI-fertilized blastocysts. An explanation for these findings is that in many cycles, the IVF-fertilized embryos were further ahead in their development than were the ICSI-fertilized embryos, and were therefore chosen for transfer. Shoukir et al. [17] also found that the rate of blastocyst development was significantly higher (47.3%) in IVF-derived embryos than the rate in ICSI-derived embryos (26.8%). They state that the embryos obtained by IVF fertilization were of significantly better quality than the embryos obtained by ICSI fertilization. In their discussion, the authors review problems that may arise when abnormal spermatozoa enter oocytes by the process of ICSI.

It might be expected that if the IVF-fertilized embryos had a tendency to be more rapid in their development than the ICSI-fertilized embryos, then the IVF-fertilized embryos might also result in a higher percentage of livebirths, but instead, the ICSI-fertilized embryos resulted in a non-significantly higher percentage of livebirth events than the IVF-fertilized embryos. It is interesting that two of the ICSI-derived and one of the IVF-derived twin livebirths in Table 6 each resulted from the transfer of two cleavage-stage embryos. These observations indicate that although ICSI-fertilized embryos may have been slower to develop than the corresponding IVF-fertilized embryos, once they had reached a certain stage of development, they were competent in proceeding to implantation and livebirth. Exactly what events in its development give an embryo the ability to proceed to livebirth after transfer to the uterus is uncertain. The 3 pairs of cleavage stage embryos in the IVF-derived and ICSI-derived columns that gave rise to 3 twin livebirths in this study comprised one 6-cell, four 8-cell and one 9-cell embryo. This suggests that at least 6 cells in the embryo may be obligatory for livebirth to result but 8 cells give a better likelihood.

The finding that blastocyst transfer is usually more successful than the transfer of embryos at earlier stages of development [3, 10] was confirmed in this study. Mixed blastocysts comprised only 57.4% of total mixed transferred embryos (Table 4), whereas the percentage of blastocysts in IVF-derived and ICSI-derived transferred embryos was 85.1 and 77.3% respectively. The significance of the difference between 57.4% and the IVF and ICSI percentages is < 0.001 and 0.003 respectively. The significantly smaller percentage of blastocysts in the mixed transferred embryos compared to the IVF-derived and ICSI-derived embryos could explain the lower percentage of livebirth events from the mixed embryos seen in Table 5. The 20 cleavage stage mixed embryos resulted in no twin livebirths and only 2 of the 10 singleton livebirths in the mixed column. A pair of pre-morulas resulted in one singleton livebirth, and the remaining 7 singleton livebirths all resulted from blastocysts.

The advent of ICSI led to a number of studies comparing post-fertilization outcomes from IVF- and ICSI-fertilized embryos, some of which used sibling oocytes in split cycles as in the present study. Two reports, Chiamchanya et al. [7] (who used IVF at higher than the usual sperm concentration) and Shveiky et al. [18] used split cycles, and both studies found no significant difference in implantation rate or pregnancy rate between IVF-derived and ICSI-derived embryos. Staessen et al. [20], working with IVF/ICSI split cycles of 47 couples, record that 42 h after fertilization, more ICSI-fertilized embryos than IVF-fertilized embryos had reached the 4-cell stage (*P* < 0.001) and more IVF-fertilized embryos than ICSI-fertilized embryos were left behind at the 2-cell stage (*P* < 0.02). Lemmen et al. [15] using a time-lapse monitoring system supported this observation. The faster development of ICSI-derived embryos is reported to last until the 5-cell [20] or 6-cell [13] stage.

The evidence described in our present study, which indicates that IVF-derived embryos develop faster than ICSI-derived embryos to the blastocyst stage is based on the significantly greater number of IVF-derived blastocysts compared to ICSI-derived blastocysts on the day when blastocysts were first seen. This difference in development rate resulted in a greater number of IVF-derived embryos available for transfer. This situation applies to the later phase of embryo development when the embryo has ceased to rely upon maternal transcripts and is fully controlled by its own genome. This happens at a time between the four-cell and eight-cell embryo stages [4, 21].

Many research studies have compared the two fertilization methods having one set of patients for fertilization by IVF and another set of patients, perhaps with poorer quality sperm, for fertilization by ICSI. Care is needed in the interpretation of this type of study, and it is important that the oocytes destined for ICSI are allowed time in contact with their cumulus cells before denudation [23]. These studies can yield useful information however. Dumoulin et al. [8] carried out the widely used procedure of fertilizing couples with poor sperm by ICSI and couples with good-quality sperm by IVF. They found that fertilization by injection with poor-quality sperm had no effect on embryo development up to day 3 after fertilization (the time of reliance on maternal transcripts). However, at later stages of development, when the embryonic genome is in control, the original injection with poor-quality sperm had a marked adverse effect on embryo development. IVF-fertilized embryos would probably be affected in the same way if an inferior spermatozoon had previously entered the oocyte. However, in the case of IVF, competition between spermatozoa exists, and better-quality spermatozoa are more likely to achieve fertilization of an oocyte than those of lower quality. Thus even in split cycles, where the same sperm sample is used for the IVF and ICSI fertilizations, the IVF-fertilized embryos are more likely to receive a better quality spermatozoon, and their developmental progress toward blastocyst formation would therefore proceed more rapidly than that of the ICSI-fertilized embryos. The faster development rate of the IVF-fertilized embryos might simply be a matter of whether the spermatozoon is chosen by natural events or by human participation.

This theory, that IVF-derived embryos are fertilized by sperm superior to those fertilizing ICSI, has an advantage over any attempt to explain the slower rate of blastocyst formation by ICSI on proposed delays related to the ICSI process. The superior-sperm theory explains why ICSI-derived embryos start their development faster than IVF-derived embryos and only become slower at the 5–6-cell stage when the male genes fully participate in the genome [4, 13, 15, 20].

In this study, we found no significant difference between IVF and ICSI in either fertilization rate or livebirth rate. However, the success of ICSI in equalling or exceeding the livebirth rate from IVF, and (an observation external to this paper) achieving livebirths when sperm progressive motility is close to zero, draws attention away from possible or likely infertility in male offspring resulting from its use. The continuing use of IVF where indicated by the history, diagnosis and general situation of the infertile couple is justified by considerations of convenience and expense and, most importantly, by its superiority over ICSI in bequeathing ongoing fertility to future generations.
